# Effects of Vitamin D Supplementation on Surrogate Markers of Fertility in PCOS Women: A Randomized Controlled Trial

**DOI:** 10.3390/nu13020547

**Published:** 2021-02-07

**Authors:** Elisabeth Lerchbaum, Verena Theiler-Schwetz, Martina Kollmann, Monika Wölfler, Stefan Pilz, Barbara Obermayer-Pietsch, Christian Trummer

**Affiliations:** 1 Department of Internal Medicine, Division of Endocrinology and Diabetology, Medical University of Graz, Auenbruggerplatz 15, 8036 Graz, Austria; verena.schwetz@medunigraz.at (V.T.-S.); stefan.pilz@medunigraz.at (S.P.); barbara.obermayer@medunigraz.at (B.O.-P.); christian.trummer@medunigraz.at (C.T.); 2 Department of Obstetrics and Gynecology, Division of Gynecological Endocrinology and Reproductive Medicine, Medical University of Graz, Auenbruggerplatz 14, 8036 Graz, Austria; martina.kollmann@medunigraz.at (M.K.); monika.woelfler@medunigraz.at (M.W.)

**Keywords:** vitamin D, polycystic ovary syndrome, anti-Müllerian hormone, follicle-stimulating hormone, randomized controlled trial

## Abstract

Vitamin D (VD) might play an important role in polycystic ovary syndrome (PCOS) and female fertility. However, evidence from randomized controlled trials (RCT) is sparse. We examined VD effects on anti-Müllerian hormone (AMH) and other endocrine markers in PCOS and non-PCOS women. This is a post hoc analysis of a single-center, double-blind RCT conducted between December 2011 and October 2017 at the endocrine outpatient clinic at the Medical University of Graz, Austria. We included 180 PCOS women and 150 non-PCOS women with serum 25-hydroxyvitamin D (25(OH)D) concentrations <75 nmol/L in the trial. We randomized subjects to receive 20,000 IU of VD3/week (119 PCOS, 99 non-PCOS women) or placebo (61 PCOS, 51 non-PCOS women) for 24 weeks. Outcome measures were AMH, follicle-stimulating hormone (FSH), luteinizing hormone (LH), estradiol, dehydroepiandrosterone sulfate, and androstenedione. In PCOS women, we observed a significant treatment effect on FSH (mean treatment effect 0.94, 95% confidence interval [CI] 0.087 to 1.799, *p* = 0.031) and LH/FSH ratio (mean treatment effect −0.335, 95% CI −0.621 to 0.050, *p* = 0.022), whereas no significant effect was observed in non-PCOS women. In PCOS women, VD treatment for 24 weeks had a significant effect on FSH and LH/FSH ratio but no effect on AMH levels.

## 1. Introduction

Vitamin D (VD) is a steroid hormone with well-known effects on calcium and bone metabolism [1]. Accumulating evidence from cross-sectional studies indicates an association of low 25-hydroxyvitamin D (25(OH)D) concentrations with various conditions including obesity, metabolic disorders [2,3], cardiovascular disease [4], hypogonadism [5], polycystic ovary syndrome (PCOS) [6], and decreased female fertility [7]. It has been hypothesized that a possible VD effect on ovarian anti-Müllerian hormone (AMH) might be a putative component explaining the complex relationship of VD and human reproduction [8]. AMH is an ovarian biomarker playing a central role in folliculogenesis and ovarian dysfunction. Several in vitro as well as in vivo studies examined the potential effects of vitamin D on ovarian function [9,10]. Kinuta et al. [9] found that VD receptor null female mice suffer from ovarian insufficiency that is characterized by impaired follicular development. A recent meta-analysis assessed the reproductive outcomes of 2700 subfertile women and found a significant association of favorable outcomes with replete vitamin D status [10]. It has been hypothesized that VD acts upon the ovarian follicle and may improve oocyte quality [10]. As impaired ovarian function is also related to obesity, it should be mentioned that obesity is associated with low VD status due to decreased physical activity, low sun exposure, and sequestration in the adipose tissue [11,12]. Furthermore, it has been hypothesized that low 25(OH)D concentrations are involved in the development of obesity by influencing adipogenesis [12].

PCOS is the most common endocrine disorder among women of childbearing age [13]. Of note, PCOS has a very high prevalence and up to 10% of women of reproductive age are affected by PCOS [13]. In addition to hyperandrogenemia and metabolic disturbances such as obesity and insulin resistance, affected women frequently suffer from decreased fertility due to anovulation [13,14]. Moreover, alterations in lipid pattern are associated with obstetric complications in PCOS women [15]. Diet plays an important role in the pathogenesis of PCOS and obesity is related to the severity of the syndrome [16,17]. An increasing number of studies have examined the association of VD status with various features of PCOS. Whereas the majority of observational studies point towards a link of deficient VD status with obesity, metabolic disturbances, and anovulation, data derived from randomized controlled trials (RCTs) are limited [6]. Compared to healthy women, PCOS women have higher AMH levels and AMH is considered as an important diagnostic and prognostic marker in PCOS [18]. Existing cross-sectional studies on the association of 25(OH)D concentrations and AMH levels have reported inconsistent results [19]. Although a small RCT among VD-deficient infertile PCOS women reported a positive VD effect on AMH levels [20], data from large RCTs are lacking. Therefore, a recent systematic review and meta-analysis on VD and AMH concluded that large RCTs of VD supplementation are necessary to elucidate the complex relationship of VD and AMH [19].

Consequently, we performed a post hoc analysis of our RCT that was designed to examine VD effects on endocrine and metabolic parameters in PCOS and non-PCOS women. We aim to investigate VD effects on AMH levels as well as on other endocrine parameters involved in reproduction, including follicle-stimulating hormone (FSH), luteinizing hormone (LH), and estradiol in PCOS as well as in healthy premenopausal women without PCOS. Furthermore, we analyze VD effects on dehydroepiandrosterone sulfate (DHEAS) and androstenedione levels.

## 2. Materials and Methods

This study is a post hoc analysis of a single-center, placebo-controlled, double-blind, parallel-group study performed at the Medical University of Graz (MUG), Austria. We designed our study to examine VD effects on endocrine and metabolic parameters in PCOS as well as in healthy women without PCOS.

We have published details on the study design and methods previously [21,22]. The design, conducting, and publication of this study adhere to the recommendations of the CONSORT Statement (http://www.consort-statement.org/). We registered the trial at http://www.clinicaltrialsregister.eu (EudraCT number, 2011-000994-30) and at clinicaltrials.gov (ClinicalTrials.gov Identifier NCT01721915). The local ethics committee approved the study protocol (EK 23-300 ex 10/11).

### 2.1. Subjects

Premenopausal women aged ≥18 years with 25(OH)D concentrations <75 nmol/L were eligible for our study. In the PCOS group, we established a diagnosis of PCOS using the Rotterdam criteria [23] if two out of the following three features were met: clinical and/or biochemical hyperandrogenism, polycystic ovaries, and/or oligo-/anovulation. We excluded disorders with similar clinical features before we made the diagnosis of PCOS. Non-PCOS women were required to show none of the Rotterdam PCOS criteria.

Exclusion criteria in both groups were hypercalcemia (defined as plasma calcium concentrations >2.65 mmol/L), regular vitamin D supplementation within 3 months prior to study inclusion, prevalent type 2 diabetes mellitus, use of insulin-sensitizing drugs (i.e., metformin, incretin mimetic drugs, thiazolidinedione, sulfonylurea) within 6 months prior to study inclusion, hormonal contraception within 3 months prior to study inclusion, use of lipid-lowering drugs or other drugs affecting insulin sensitivity or serum androgens (e.g., niacin, corticosteroids, beta-blockers, calcium channel blockers, thiazide diuretics) as well as disorders apart from PCOS associated with irregular menses and/or androgen excess.

We recruited PCOS and non-PCOS women from patients of the Division of Endocrinology and Diabetology and the Division of Gynecological Endocrinology and Reproductive Medicine at the MUG by conversation during routine visits in the outpatient clinics. Furthermore, we recruited participants from female hospital staff and female family members of hospital staff, and written information about the study was posted in the outpatient clinic. We informed all study participants during recruitment about the possibility of receiving a placebo.

#### Healthy Women

We included not only PCOS but also healthy women in our RCT as vitamin D might have varying effects among women with and without PCOS. As outlined above, the relationship of vitamin D and AMH is complex. Vitamin D might increase AMH levels in healthy women [24] but decrease AMH levels in PCOS women [20]. Therefore, to examine whether vitamin D effects vary depending on the respective group, we included PCOS as well as healthy women without PCOS in our analyses.

### 2.2. Intervention

We allocated subjects to the placebo (PBO) or VD group according to a computer-generated randomization list using a ratio of 2:1. We placed study medication into numbered bottles according to this list.

The VD group received an oral dose of 20,000 IU VD3 per week (equivalent to 2857 IU/day) as 50 oily drops per week (Oleovit D3 drops; Fresenius Kabi Austria GmbH, Linz) for 24 weeks. Our PBO group received 50 oily drops without VD for 24 weeks. PBO oil contained the same oil as Oleovit D3 drops (without VD content). Fresenius Kabi Austria GmbH, Linz delivered the PBO oil. All investigators involved in the enrollment of study subjects, data collection as well as assignment to intervention were masked to participant allocation. In order to improve and verify compliance, we asked study participants to return full as well as empty study medication bottles at the end of the study.

### 2.3. Outcome Measures

This is a post hoc analysis of our RCT including PCOS and non-PCOS women. We investigate VD effects on endocrine parameters including AMH, FSH, LH, estradiol, DHEAS, and androstenedione levels.

### 2.4. Procedures

We collected basal blood samples for measurement of 25(OH)D, AMH, FSH, LH, estradiol, DHEAS, and androstenedione between 8.00 and 9.00 a.m. after an overnight fast. We used 25(OH)D concentrations determined by immunoassay for evaluation of inclusion criteria. We performed biobanking of all remaining blood samples by freezing and storing at −80 °C until analysis. In addition, we measured serum concentrations of 25(OH)D by well-adjusted isotope dilution–liquid chromatography tandem mass spectrometry (ID-LC-MS/MS) methods in 2018 [21,22].

We measured FSH, LH, and estradiol levels on a daily basis. LH and FSH were measured using Access^®^ hLH and hFSH CLIA (Beckman Coulter Inc., Brea, CA, USA), respectively. 17β-estradiol was determined using IMMULATE^®^ CLIA assays (Siemens Healthcare Diagnostics Products Ltd., Glyn Rhonwy, UK). We measured AMH, androstenedione, and DHEAS levels once weekly, and blood samples were frozen and stored at −40 °C until analysis. We measured DHEAS (Labor Diagnostika Nord, Nordhorn, Germany) and androstenedione (Siemens Healthcare Diagnostics Products Ltd.) via enzyme-linked immunosorbent assay (ELISA), with intra-assay and interassay coefficients of variation (CV) of <10%. In our laboratory, the assay for AMH was changed in November 2014 from the ultra-sensitive anti-Müllerian hormone/Müllerian-inhibiting substance enzyme-linked immunosorbent assay (ELISA) kit (Ansh Labs, Webster, TX, USA) to the Access 2 immunosorbent assay system (Beckmann Coulter). We compared both assays and found a good correlation (r = 0.95). Both AMH assays show intra-assay and interassay CV of <10%. Laboratory kits and assays did not change between 2011 and 2017 for the remaining outcome parameters.

Vitamin D intake was assessed by questionnaires.

### 2.5. Statistical Analyses

We performed sample size calculation based on the data derived from a pilot study conducted among PCOS women [25]. In detail, we found a reduction area under the curve (AUC) glucose from 115 ± 17 at baseline to 103 ± 18 at the end of the study after 24 weeks VD supplementation. We therefore calculated a sample size of 92 participants to detect a treatment difference at two-sided 0.05 significance levels with a probability of 90%, if the true difference between treatments is 12 with a standard deviation of 17. As the analyses of VD effects according to genotype profile were a secondary outcome measure (results have been published previously [26]), we randomized study participants 2:1 (VD:PBO) in order to increase the sample size in the VD group. The number of enrolled PCOS subjects was increased from 150 to 180 to ensure an adequate power to detect differences regarding AUCgluc.

We used descriptive statistics as well as the Kolmogorov–Smirnov test to analyze the distribution of data. We present continuous data with normal distribution as means with SD and continuous data following a skewed distribution as median with interquartile range. We performed log transformation of skewed variables and rechecked log transformed data for normal distribution before parametric tests were performed. We used Student’s T-test and χ^2^-test for comparisons of baseline characteristics between groups. Delta (Δ) values (value at the end of the study minus baseline value) were calculated for 25(OH)D and outcome measures. We used Pearson correlation analysis to determine relationships between variables. We performed multivariable stepwise linear regression analysis with LH/FSH ratio and androstenedione as the dependent variables, and with BMI, age, and 25(OH)D as independent variables.

We executed analyses of outcome variables according to the intention-to-treat principle. In these analyses, we included all subjects with baseline and follow-up values. We applied analysis of covariance and adjusted our analyses for baseline values to test for differences in the respective outcome variables between the VD and the PBO group at the end of the study. We performed subgroup analyses of PCOS women with irregular menses. All statistical procedures were performed with SPSS version 26 (SPSS Inc., Chicago, IL, USA). We considered a *p*-value of <0.05 as statistically significant.

## 3. Results

We screened ~500 PCOS women and ~300 healthy women without PCOS who routinely visited the endocrine outpatient clinic or responded to written information material for study eligibility. We randomized and enrolled 180 PCOS women and 150 healthy women in the study (participant flow charts have been published previously [21,22]).We randomized the first subject in December 2011 and we performed the last follow-up in October 2017.

In Table 1, we display the baseline characteristics of all study subjects. In PCOS women, we observed significantly higher BMI (*p* = 0.001), AMH levels (*p* < 0.001), LH levels (*p* = 0.02), LH/FSH ratio (*p* < 0.001), DHEAS (*p* < 0.001), and androstenedione levels (*p* < 0.001), whereas age (*p* < 0.001), 25(OH)D (*p* = 0.019), FSH (*p* < 0.001), and estradiol levels (*p* < 0.001) were lower compared to healthy women without PCOS. PCOS women in the VD group were significantly younger compared to PCOS women in the PBO group. In healthy women without PCOS, baseline estradiol levels were significantly lower in the VD group compared to the PBO group. We found no significant differences among the remaining baseline characteristics between VD and PBO groups in PCOS as well as in healthy women.

### 3.1. Cross-Sectional Analyses

In PCOS women, we found a significant correlation of 25(OH)D levels with LH/FSH ratio (*r* = −0.195, *p* = 0.009) as well as with androstenedione levels (*r* = 0.15, *p* = 0.043). We observed no significant correlation of 25(OH)D levels with the remaining endocrine parameters (AMH, LH, FSH, estradiol, and DHEAS). In analyses adjusted for age and BMI, the correlation of 25(OH)D with LH/FSH ratio (*p* = 0.011) remained stable but was attenuated for androstenedione (*p* = 0.070).

In healthy women, we observed no significant correlation of 25(OH)D with endocrine parameters.

### 3.2. Outcome Analyses

#### 3.2.1. PCOS Women

In PCOS women, the mean (±SD) overall treatment period was 176 ± 23 days in the VD group and 176 ± 21 days in the PBO group (*p* = 0.906). A total of 123 study participants completed both study visits.

In Table 2, we display results of outcome analyses. In PCOS women, we found a significant VD effect on FSH levels as well as on LH/FSH ratio. We found no significant effect on the remaining outcome parameters. After exclusion of PCOS women with regular menses (*n* = 19), VD effects on FSH levels (mean treatment effect 0.271, 95% CI 0.27 to 2.06, *p* = 0.011) and LH/FSH ratio (mean treatment effect −0.401, 95% CI −0.705 to −0.097, *p* = 0.010) remained stable.

We observed a significant negative correlation of Δ25(OH)D levels with ΔLH/FSH ratio (*r* = −0.208, *p* = 0.024) and a trend with ΔFSH (*r* = 0.169, *p* = 0.066). We observed no significant correlation of Δ25(OH)D with ΔAMH, ΔLH, Δestradiol, Δandrostenedione, and ΔDHEAS (*p* > 0.05 for all).

Table 3 shows 25(OH)D concentrations at baseline and the end of the study in PCOS women. VD supplementation significantly increased 25(OH)D concentrations.

VD effects on metabolic parameters are shown in supplemental Tables S1 and S2. In PCOS women, we found a significant beneficial VD effect on glucose levels at 60 min during the oral glucose tolerance test (Supplementary Table S1). In non-PCOS women, VD treatment had a significant unfavorable effect on insulin resistance and insulin sensitivity (Supplementary Table S2).

#### 3.2.2. Non-PCOS Women

In healthy women, the mean (± SD) treatment duration was 174 ± 44 days in the VD group and 173 ± 23 days in the PBO group (*p* = 0.884). In total, 127 participants completed the entire study including the last follow-up visit after 24 weeks.

In healthy women without PCOS, we found no significant VD effect on outcome measures (*p* > 0.05 for all, data not shown). Furthermore, we observed no significant correlation of Δ25(OH)D with changes in outcome measures (*p* > 0.05 for all).

Table 3 shows 25(OH)D concentrations at baseline and the end of the study in healthy women. We found a significant VD effect on 25(OH)D concentrations in women without PCOS.

## 4. Discussion

In our RCT in PCOS women with baseline 25(OH)D concentrations <75 nmol/L, VD treatment had a significant effect on FSH levels and LH/FSH ratio. We found, however, no significant VD effect on AMH levels and the remaining endocrine parameters. In healthy women with serum 25(OH)D <75 nmol/L at baseline, we observed no significant VD effect on outcome measures.

Interestingly, we observed a significant VD effect on FSH levels and LH/FSH ratio as well as a significant correlation between Δ25(OH)D and ΔLH/FSH ratio in PCOS women. In the pathophysiology of PCOS, abnormalities of the hypothalamic–pituitary–ovarian axis play an important role [13]. A relative increase in LH to FSH release is caused by a disturbance in the secretion pattern of the gonadotrophin-releasing hormone [27]. Furthermore, ovarian estrogen is responsible for causing an abnormal feedback mechanism that results in increased LH release [28]. An elevated LH/FSH ratio is a common finding in PCOS and as a result, ovulation does not occur in many PCOS patients [29]. It has been reported that VD alters FSH sensitivity, indicating a possible physiological role for VD in the development and luteinization of the ovarian follicle [8]. Among induced PCOS rats, VD treatment increases the normal follicle number through increasing FSH and estradiol and decreasing LH [30]. Furthermore, Kinuta et al. [9] demonstrated that VD promoted folliculogenesis and follicular development in PCOS rats by increasing progesterone and estrogen levels and regulating the LH/FSH ratio.

Our results contribute to the mounting evidence from cross-sectional and interventional studies on favorable VD effects on reproduction [7,31,32]. It has been hypothesized that physiological levels of VD might have a beneficial role in ovulation and endometrial receptivity [33]. Consistently, findings from systematic reviews and meta-analyses analyzing the association of VD and assisted reproduction outcomes suggest that women with replete VD status have more live births, more positive pregnancy tests, and more clinical pregnancies compared to women with deficient or insufficient 25(OH)D [31,32]. Recently, Butts et al. [31] reported that VD deficiency in PCOS women who underwent ovarian stimulation for infertility treatment was linked with significantly diminished rates of ovulation, of pregnancy, and ultimately, a reduced chance of live birth. Of note, there was no significant association of VD deficiency with ovulation, pregnancy, or live birth in non-PCOS women with unexplained infertility [31]. In light of the high prevalence of insufficient VD levels in PCOS women [6] and the significant burden of decreased fertility in affected women, our findings deserve investigation in future large RCTs including PCOS women as well as women without PCOS. Considering the fact that VD supplementation is a safe and cheap treatment, our findings might be of high clinical interest. It should, however, be emphasized that the clinical relevance of our findings regarding reproduction remains to be determined as we investigated only surrogate parameters involved in fertility.

We failed to find a significant VD effect on AMH levels. Existing evidence on the relationship of VD and AMH levels is conflicting [19]. It has been shown that VD regulates AMH levels in vitro, both directly through the AMH promoter [34] and indirectly by regulating the number of granulosa cells and AMH signaling in cultures of ovarian follicles [35]. In contrast to the consistency of the in vitro data, the evidence of a link between VD and AMH in women is contentious. The majority of cross-sectional studies failed to find a significant correlation of 25(OH)D levels and AMH [19]. In contrast, a prospective study including PCOS women observed an association of VD supplementation with a decrease in serum AMH levels [36]. Furthermore, positive VD effects on AMH levels were found in a prospective study including infertile women with diminished ovarian reserve [24]. To date, there are only two small RCTs investigating VD effects on AMH levels in women [20,37]. In a study among VD-deficient infertile PCOS women, participants received either 50,000 IU VD/week (*n* = 17) or PBO (*n* = 17) for 8 weeks. The authors found a significant decrease in AMH levels in the VD group compared with PBO [20]. Dennis et al. [37] conducted an RCT in 49 young women with regular menses to evaluate the effects of a single high dose of VD (50,000 IU, taken on the first day of the menstrual cycle) versus PBO on AMH levels during the following week. Interestingly, the authors observed a significant progressive increase in AMH levels in the following week after VD supplementation. In our RCT, we found no significant VD effect on AMH levels in PCOS or non-PCOS women. These different results might be related to varying VD doses, study duration, baseline 25(OH)D levels, age, and sample size. However, as 25(OH)D concentrations at the end of the study were high in both groups, it is unlikely that the lack of a significant VD effect on AMH levels in our study is related to insufficient vitamin D doses.

Our study has several limitations. First, as we investigated women with relatively high baseline 25(OH)D levels, we cannot exclude significant VD effects on AMH levels in women with lower baseline 25(OH)D levels. Another possible limitation is the relatively high drop-out rate in PCOS women. Furthermore, since blood samples were collected regardless of the participants’ menstrual cycle, the results regarding some of the measured parameters (e.g., FSH, LH, estradiol) should be interpreted with caution. As gonadotropins vary consistently during the phases of the menstrual cycle, our results regarding vitamin D effects on FSH and LH/FSH ratio should be interpreted in light of this limitation. We cannot rule out that blood sampling in the first week of the menstrual cycle in PCOS women with a regular menstrual cycle would provide different results. Nevertheless, only a small number of PCOS women had a regular menstrual cycle (*n* = 19) and the exclusion of these PCOS women from our analyses did not materially change our results. Moreover, AMH levels are stable across the menstrual cycle and typically demonstrate minimal intercycle and intracycle variability [38,39]. As we did not assess data on sun exposure, we were not able to adjust our analyses for this potential confounder. Furthermore, we did not assess the dietary pattern of study participants. This limitation might influence our results, as it has been demonstrated that specific diets such as the Mediterranean diet are associated with circulating 25(OH)D concentrations [40]. Finally, our findings should be interpreted with caution because our results derive from a post hoc analysis and we did not adjust for multiple testing as our analyses were all based on a priori pre-specified hypotheses. Nevertheless, we cannot exclude that our statistical analyses revealed a false-positive finding. The strengths of our study include its design as an RCT, the large sample size as well as the inclusion of PCOS and non-PCOS women.

## 5. Conclusions

In summary, we found no significant VD effect on AMH levels but a significant effect on FSH levels and LH/FSH ratio in PCOS women. Our results, therefore, support the idea that VD may be involved in reproductive function in PCOS women. In light of previous data suggesting a possible favorable VD effect on female fertility, further adequately powered RCTs are of clinical importance to clarify the potential positive effects of VD on reproductive function in PCOS women.

## Figures and Tables

**Table 1:** Baseline characteristics of study subjects. Data are shown as means with standard deviation, median, and interquartile range or proportion as appropriate. PCOS—polycystic ovary syndrome; VD—vitamin D; PBO—placebo; BMI—body mass index; 25(OH)D—25-hydroxyvitamin D; AMH—anti-Müllerian hormone; FSH—follicle-stimulating hormone; LH—luteinizing hormone; DHEAS—dehydroepiandrosterone sulfate. We performed comparisons of baseline characteristics between women in the VD and the PBO groups using Student’s *t*-test and χ^2^-test. Season 1: January–March; season 2: April–June; season 3: July–September; season 4: October–December.

	**All PCOS Women** **(*n* = 180)**	**VD (*n* = 119)**	**PBO (*n* = 61)**	***p*-Value**
Age (years)	26.0 ± 5.0	25.4 ± 4.6	27.2 ± 5.5	0.022
BMI (kg/m^2^)	27.6 ± 7.5	27.3 ± 7.4	28.3 ± 7.8	0.453
25(OH)D * (nmol/L)	50.4 ± 19.0	50.7 ± 19.5	49.9 ± 18.3	0.798
AMH (ng/mL)	7.67 (4.09–15.0)	7.62 (4.23–15.0)	7.71 (3.15–15.0)	0.547
FSH (µU/mL)	5.97 ± 2.41	6.04 ± 2.59	5.94 ± 2.33	0.783
LH (µU/mL)	8.88 (4.26–14.5)	8.89 (4.20–15.34)	8.86 (3.82–14.18)	0.830
LH/FSH ratio	1.48 (0.88–2.30)	1.52 (0.88–2.54)	1.38 (0.68–2.55)	0.530
Estradiol (pg/mL)	60.6 (41.0–122.0)	59.1 (39.3–123.0)	64.0 (43.5–158.0)	0.311
DHEAS (µg/mL)	1.90 (1.24–2.97)	1.94 (1.16–3.22)	1.9 (1.28–3.07)	0.789
Androstenedione (ng/mL)	3.36 (2.26–4.87)	2.4 (1.48–4.24)	2.61 (1.79–3.96)	0.937
Vitamin D intake (IU/day)	31 (14–76)	31 (16–67)	31 (13–78)	0.582
Season of recruitment				
Season 1	38.3%	36.1%	42.6%	0.442
Season 2	26.1%	26.1%	26.2%	
Season 3	17.8%	21.0%	11.5%	
Season 4	17.8%	16.8%	19.7%	
	**All Healthy Women** **(*n* = 150)**	**VD (*n* = 99)**	**PBO (*n* = 51)**	
Age (years)	35.8 ± 8.7	35.7 ± 8.9	36.1 ± 8.4	0.826
BMI (kg/m^2^)	25.2 ± 5.5	25.5 ± 5.3	24.7 ± 5.8	0.398
25(OH)D * (nmol/L)	55.4 ± 18.9	55.4 ± 18.9	55.3 ± 18.9	0.996
AMH (ng/mL)	1.97 (0.32–4.38)	1.89 (0.29–5.2)	2.41 (0.32–5.30)	0.546
FSH (µU/mL)	9.86 ± 13.11	9.67 ± 12.05	9.96 ± 13.69	0.898
LH (µU/mL)	6.28 (3.72–11.0)	6.28 (3.24–11.50)	6.48 (4.04–14.20)	0.119
LH/FSH ratio	0.93 (0.51–1.59)	0.87 (0.48–1.57)	1.12 (10.51–2.03)	0.242
Estradiol (pg/mL)	92.6 (50.5–156.0)	83.4 (41.5–145)	114 (61.1–212.0)	0.006
DHEAS (µg/mL)	1.21 (0.78–2.0)	1.20 (0.75–2.03)	1.23 (0.76–2.19)	0.508
Androstenedione (ng/mL)	2.50 (1.56–3.96)	2.4 (1.48–4.24)	2.61 (1.79–3.96)	0.642
Vitamin D intake (IU/day)	50 (26–77)	50 (22–80)	50 (27–72)	0.471
Season of recruitment				
Season 1	30.7%	29.3%	33.3%	0.942
Season 2	32.7%	32.3%	33.3%	
Season 3	10.0%	10.1%	9.8%	
Season 4	26.7%	28.3%	23.5%	

* We measured 25(OH)D by liquid chromatography tandem mass spectrometry.

**Table 2:** Continuous outcome variables at baseline and end of the study in PCOS women with available values at both study visits. We display data as means with standard deviation or medians and interquartile range as appropriate. We calculated treatment effects with 95% confidence interval and *p*-values by analysis of covariance for group differences at the end of the study. Analyses were adjusted for baseline values. IQR—interquartile range; AMH—anti-Müllerian hormone; VD—vitamin D; PBO—placebo; FSH—follicle-stimulating hormone; LH—luteinizing hormone; DHEAS—dehydroepiandrosterone sulfate.

	Baseline Visit	Study End	Treatment Effect (95% Confidence Interval)	*p*-Value
AMH * (ng/mL)				
VD (*n* = 80)	7.6 (4.2–15.0)	7.0 (4.2–15.5)	0.097 (−0.081 to 0.276)	0.282
PBO (*n* = 40)	7.7 (3.2–15.0)	7.6 (2.8–14.4)		
FSH (µU/mL)				
VD (*n* = 81)	6.04 ± 2.59	6.16 ± 2.46	0.94 (0.087 to 1.799)	0.031
PBO (*n* = 41)	5.94 ± 2.33	5.23 ± 1.78		
LH * (µU/mL)				
VD (*n* = 79)	8.9 (4.2–15.3)	9.4 (3.4–15.2)	−0.184 (−0.497 to 0.129)	0.248
PBO (*n* = 41)	8.9 (3.8–14.2)	8.8 (4.1–14.7)		
Estradiol * (pg/mL)				
VD (*n* = 81)	59.1 (39.3–123.0)	59.4 (33.9–169.0)	−0.096 (−0.351 to 0.159)	0.460
PBO (*n* = 41)	64.0 (43.5–158.0)	73.8 (44.2–193.0)		
LH/FSH ratio *				
VD (*n* = 79)	1.52 (0.88–2.54)	1.45 (0.79–2.73)	−0.335 (−0.621 to −0.050)	0.022
PBO (*n* = 41)	1.38 (0.69–2.55)	1.73 (0.76–3.32)		
DHEAS * (µg/mL)				
VD (*n* = 81)	1.94 (1.16–3.22)	1.96 (1.06–3.12)	−0.016 (−0.142 to 0.11)	0.805
PBO (*n* = 41)	1.9 (1.28–3.07)	2.12 (1.31–3.23)		
Androstenedione * (ng/mL)				
VD (*n* = 80)	3.41 (2.24–4.95)	3.68 (2.55–6.0)	0 (−0.131 to 0.130)	0.996
PBO (*n* = 40)	3.32 (2.05–5.58)	3.86 (2.33–7.11)		

* Skewed variables for which logarithmic transformed values were used in ANCOVA, but untransformed values are shown in the table.

**Table 3:** 25(OH)D concentrations at baseline and at the end of the study in subjects with available values at both study visits. Data are shown as means with standard deviation. Treatment effects with 95% confidence interval and *p*-values were calculated by ANCOVA for group differences at follow-up with adjustment for baseline value.

	Baseline	Follow-Up (24 Weeks)	Treatment Effect (95% Confidence Interval)	*p*-Value
**PCOS women**				
25(OH)D (nmol/L)				
VD (*n* = 79)	48.8 ± 16.8	90.2 ± 20.1	33.4 (24.5 to 42.2)	<0.001
PBO (*n* = 44)	48.8 ± 17.5	56.8 ± 29.5		
**Healthy women**				
25(OH)D (nmol/L)				
VD (*n* = 82)	55.8 ± 19.9	95.3 ± 26.2	28.5 (19.3 to 37.7)	<0.001
PBO (*n* = 44)	56.2 ± 19.3	67.0 ± 24.8		

PCOS—polycystic ovary syndrome; 25(OH)D—25-hydroxyvitamin D; VD—vitamin D; PBO—placebo.

## Data Availability

The data presented in this study are available on request from the corresponding author.

## Supplementary Materials

The following are available online at https://www.mdpi.com/2072-6643/13/2/547/s1, Table S1: Outcome variables at baseline and follow-up at study end in PCOS women with available values at both study visits, Table S2: Outcome variables at baseline and follow-up at study end in non-PCOS women with available values at both study visits.
