# Supplementary material for: Effects of Vitamin D Supplementation on Surrogate Markers of Fertility in PCOS Women: A Randomized Controlled Trial
Source: Nutrients. 2021 Feb 7;13(2):547. doi: 10.3390/nu13020547 (PMC7914670; doi:10.3390/nu13020547)
Supplement: Supplementary file 1 [file nutrients-13-00547-s001.pdf]

Supplementary Materials

Supplementary Table 1. Outcome variables at baseline and follow-up at study end in PCOS women with available values at both study visits. These data have been published previously [21].

	Baseline	Follow-Up (24 weeks)	Treatment Effect (95% CI)	p-value
	Fasting glucose [mg/dL]			
Vitamin D (n=81)	84±8	82±8	-1.2 (-3.6 to 1.3)	0.353
Placebo (n=42)	84±8	83±7		
	OGTT glucose 30 min [mg/dL]			
Vitamin D (n=80)	133±24	130±23	-1.6 (-10.0 to 6.8)	0.711
Placebo (n=42)	128±25	129±26		
	OGTT glucose 60 min [mg/dL]			
Vitamin D (n=80)	123±39	105±31	-10.2 (-20.2 to -0.3)	0.045
Placebo (n=42)	107±31	107±34		
	OGTT glucose 120 min [mg/dL]			
Vitamin D (n=81)	98±24	88±24	0.5 (-7.6 to 8.6)	0.903
Placebo (n=42)	93±24	85±24		
	*HbA1c [mmol/mol]**			
Vitamin D (n=74)	33 (31-35)	33 (32-35)	-0.4 (-0.9 to 0.2)	0.192
Placebo (n=38)	34 (32-35)	33 (32-35)		
	*HOMA-IR**			
Vitamin D (n=81)	1.95 (1.09-3.51)	2.29 (1.43-3.47)	-0.26 (-0.80 to 0.27)	0.935
Placebo (n=42)	2.15 (1.28-3.00)	2.31 (1.28-3.81)		
	*QUICKI**			
Vitamin D (n=81)	0.150 (0.138-0.164)	0.146 (0.138-0.157)	-0.002 (-0.012 to 0.008)	0.823
Placebo (n=42)	0.148 (0.141-0.160)	0.146 (0.136-0.160)		
	*Triglycerides [mg/dL]**			
Vitamin D (n=79)	62 (49-85)	71 (52-93)	3 (-7 to 12)	0.455
Placebo (n=42)	78 (50-118)	74 (48-106)		
	*Total cholesterol [mg/dL]**			
Vitamin D (n=79)	173 (158-188)	172 (158-189)	4 (-3 to 11)	0.180
Placebo (n=42)	179 (148-203)	172 (143-204)		
	AUCgluc			
Vitamin D (n=80)	226.71±46.12	208.3±39.5	-9.19 (-21.40 to 3.02)	0.139
Placebo (n=42)	213.07±40.03	208.3±42.8		

Data are shown as means with standard deviation or medians and interquartile range, as appropriate. Treatment effects with 95% confidence interval and p-values were calculated by ANCOVA for group differences at follow-up with adjustment for baseline values.

*Skewed variables for which logarithmic transformed values were used in ANCOVA, but untransformed values are shown in the table.

HbA1c = glycated hemoglobin; HOMA-IR = homeostatic model assessment-insulin resistance; OGTT glucose 30 min = plasma glucose at 30 minutes during 75g oral glucose tolerance test; OGTT glucose 60 min = plasma glucose at 60 minutes during 75g oral glucose tolerance test; OGTT glucose 120 min = plasma glucose at 120 minutes during 75g oral glucose tolerance test; QUICKI = quantitative insulin sensitivity check index, AUC = area under the curve

Supplementary table 2: Outcome variables at baseline and follow-up at study end in non-PCOS women with available values at both study visits. These data have been published previously [22].

	Baseline	Follow-Up (24 weeks)	Treatment Effect (95% CI)	p-value
		Fasting glucose [mg/dL]		
Vitamin D (n=83)	84±9	85±8	1.5 (-0.9 to 3.8)	0.214
Placebo (n=44)	86±8	84±7		
		OGTT glucose 30 min [mg/dL]		
Vitamin D (n=83)	116±28	123±33	6.9 (-2.5 to 16.3)	0.147
Placebo (n=44)	127±25	123±27		
		OGTT glucose 60 min [mg/dL]		
Vitamin D (n=82)	94±33	101±37	4.2 (-5.6 to 14.0)	0.396
Placebo (n=43)	100±33	101±33		
		OGTT glucose 120 min [mg/dL]		
Vitamin D (n=83)	83±21	84±19	3.0 (-2.8 to 8.8)	0.307
Placebo (n=44)	86±20	83±18		
		*HbA1c [mmol/mol]**		
Vitamin D (n=82)	33 (31-35)	33 (31-35)	0.2 (-0.5 to 0.9)	0.641
Placebo (n=43)	33 (31-36)	33 (32-35)		
		*HOMA-IR**		
Vitamin D (n=83)	1.77 (1.16-2.80)	1.76 (1.26-2.63)	0.31 (-0.19 to 0.74)[+]	0.019
Placebo (n=43)	1.54 (1.09-2.72)	1.42 (0.76-2.29)		
		*QUICKI**		
Vitamin D (n=83)	0.350 (0.327-0.374)	0.351 (0.330-0.371)	-0.019 (-0.033 to -0.004)	0.013
Placebo (n=43)	0.358 (0.329-0.378)	0.362 (0.337-0.402)		
		*Triglycerides [mg/dL]**		
Vitamin D (n=81)	69 (53-91)	70 (54-97)	9 (-3 to 21)	0.242
Placebo (n=42)	63 (51-73)	????		
		*Total cholesterol [mg/dL]**		
Vitamin D (n=81)	185 (160-209)	180 (163-196)	-3 (-10 to 4)	0.242
Placebo (n=42)	179 (158-193)	180 (156-206)		
		AUCgluc		
Vitamin D (n=83)	189.62±45.16	200.2±49.3	11.7 (-0.91 to 24.31)	0.069
Placebo (n=44)	201.64±41.14	197.5±45.4		

Data are shown as means with standard deviation or medians and interquartile range, as appropriate. Treatment effects with 95% confidence interval and p-values were calculated by ANCOVA for group differences at follow-up with adjustment for baseline values.

*Skewed variables for which logarithmic transformed values were used in ANCOVA, but untransformed values are shown in the table.

HbA1c = glycated hemoglobin; HOMA-IR = homeostatic model assessment-insulin resistance; OGTT glucose 30 min = plasma glucose at 30 minutes during 75g oral glucose tolerance test; OGTT glucose 60 min = plasma glucose at 60 minutes during 75g oral glucose tolerance test; OGTT glucose 120 min = plasma glucose at 120 minutes during 75g oral glucose tolerance test; QUICKI = quantitative insulin sensitivity check index, AUC = area under the curve
